# Molecular Detection of the Seed-Borne Pathogen *Colletotrichum lupini* Targeting the Hyper-Variable IGS Region of the Ribosomal Cluster

**DOI:** 10.3390/plants8070222

**Published:** 2019-07-14

**Authors:** Susanna Pecchia, Benedetta Caggiano, Daniele Da Lio, Giovanni Cafà, Gaetan Le Floch, Riccardo Baroncelli

**Affiliations:** 1 Department of Agriculture, Food and Environment, University of Pisa, Via del Borghetto 80, 56124 Pisa, Italy; 2 Laboratoire Universitaire de Biodiversité et Ecologie Microbienne, EA 3882, IBSAM, ESIAB, Université de Brest, Technopôle Brest-Iroise, 29280 Plouzané, France; 3 CABI Europe-UK, Bakeham Lane, Egham, Surrey TW20 9TY, UK; 4 Instituto Hispano-Luso de Investigaciones Agrarias (CIALE), University of Salamanca, Calle del Duero 12, 37185 Villamayor (Salamanca), Spain

**Keywords:** IGS, ribosomal intergenic spacer, *Colletotrichum acutatum*, *Lupinus*, lupins, legumes, fungal pathogens

## Abstract

Lupins anthracnose is a destructive seed and airborne disease caused by *Colletotrichum lupini*, affecting stems and pods. Primary seed infections as low as 0.01–0.1% can cause very severe yield losses. One of the most effective management strategies is the development of a robust and sensitive seed detection assay to screen seed lots before planting. PCR-based detection systems exhibit higher levels of sensitivity than conventional techniques, but when applied to seed tests they require the extraction of PCR-quality DNA from target organisms in backgrounds of saprophytic organisms and inhibitory seed-derived compounds. To overcome these limitations, a new detection protocol for *C. lupini* based on a biological enrichment step followed by a PCR assay was developed. Several enrichment protocols were compared with Yeast Malt Broth amended with ampicillin, streptomycin, and lactic acid were the most efficient. A species-specific *C. lupini* primer pair was developed based on rDNA IGS sequences. The specificity was evaluated against 17 strains of *C. lupini*, 23 different *Colletotrichum* species, and 21 different organisms isolated from seeds of *Lupinus albus* cv. Multitalia, *L. luteus* cv. Mister, and *L. angustifolius* cv. Tango. The protocol described here enabled the detection of *C. lupini* in samples artificially infected with less than 1/10,000 infected seed.

## 1. Introduction

*Lupinus* spp. are important agronomic crops worldwide, with several species that are grown as ornamentals or as pioneer plants in soil maintenance programmes [1,2]. In natural ecosystems, lupin plants are key components of a wide range of soils and climates, mainly around the Mediterranean basin, East Africa, and the entire American continent. While the species of Africa, Asia, and Europe are few and well defined, the American species are many, morphologically intersecting, and intercrossing [3].

Lupin is an important crop mainly due to its elevated seed protein content, its adaptability to low fertility soils, and its valuable influence on crop rotation [1,4,5,6]. The seed composition of specific cultivars of white lupin (*Lupinus albus*) is very similar to soybean (35% proteins and 0.5% starch), with some species that are able to reach high levels of up to 18% of oil content. The vividly colored flowers of lupins contribute to their importance as ornamental plants (e.g., *L. polyphyllus*, also known as large-leaved lupine), and are amongst the most popular ornamental plants in temperate climate areas [7]. For all these reasons, lupin production has rapidly increased in the last 10 years from 778,040 to 1,610,969 tonnes, [8]; nevertheless, effective lupin production is limited by diseases, which can lead to substantial yield and quality losses. In the past four decades, lupin production has encountered a new challenge: the devastating disease anthracnose affecting all plant tissues caused by *Colletotrichum lupini* [9,10,11,12]. 

Anthracnose has become a severe disease of lupin species worldwide, causing significant yield losses as high as 100%, representing a major limiting factor for lupin production [11,12,13,14,15]. Symptoms of anthracnose are similar for all *Lupinus* spp. [16], and are characterised by necrotic tissues with the presence of orange conidial masses and characteristic stems, petioles, and pods twisting [4]. Infected seeds become necrotic and wrinkled; when seeds are able to germinate, the seedlings show dark sunken necrosis in the cotyledons or in the hypocotyl [17]. The disease is mainly seed-borne [18], with primary seed infections as low as 0.01% that can cause extremely severe infections depending on climatic and agro ecological conditions [13,19]. While most lupin species are characterized by wide genetic diversity, it has been very difficult to find successful resistance sources. Recent achievements in this area are still limited by a very restricted number of known resistance genes, exposing lupin to the potential overcoming of such resistances [4]. 

Most species of the genus *Colletotrichum* (*Ascomycota*, *Sordariomycetes*, *Glomerellales*, and *Glomerellaceae*) are associated with plant diseases, and generally referred to as anthracnose. *Colletotrichum* spp. can infect a wide range of hosts and are distributed worldwide. Virtually every crop is susceptible to one or more species of *Colletotrichum*, which is considered one of the most important fungal plant pathogens based on scientific/economic importance [20]. Common *Colletotrichum* spp. hosts include many dicotyledonous plants and most important cereals. Species of *Colletotrichum* and *Magnaporthe* have a distinct hemibiotrophic lifestyle characterized by a brief biotrophic phase, associated with large intracellular primary hyphae and a subsequent switch to a necrotrophic phase in addition to narrow secondary hyphae that spread through the host tissue. In past decades, the genus *Colletotrichum* has been the centre of intense debate and frequent taxonomic changes [20], with recent multi-locus phylogenetics that has led to the identification of at least 14 major monophyletic clades known as “species complexes” [21,22]. *Colletotrichum* species identified within and among these complexes have shown significant differences in their host range. For example, the *C. acutatum* species complex includes *C. nymphaeae* and *C. fioriniae*, which display a broad host range, and *C. lupini*, which exhibits a host preference for lupin species. Due to this biological diversity, the *C. acutatum* species complex has been suggested as a suitable model to investigate genomic signatures associated with changes of key biological characters [4,23]. Recently, the intraspecific diversity of *Colletotrichum* associated with lupin crops in the western area of France and worldwide has been widely investigated [24]. All strains isolated from anthracnose-infected lupin have been identified as *C. lupini*, confirming that the disease is exclusively caused by this species. The genetic intraspecific diversity based on eight genomic loci with high resolution was low and revealed the presence of two distinct genetic groups [24].

The nuclear ribosomal cluster has been the most common DNA marker for PCR primer design of diagnostic assays for the detection of fungal plant pathogens. Moreover, this method allows for sensitive detection, as it is organized in units highly repeated within genomes. The cluster contains rRNA genes (18S, 5.8S, and 28S) separated by two internal transcribed spacers (ITS1 and ITS2) and one intergenic spacer (IGS) [25]. Recent phylogenetic studies have highlighted that the ITS region of the rDNA does not have sufficient variability to perform a correct taxonomic designation [26] or to develop specific primers for most *Colletotrichum* species. For this reason, other highly polymorphic single copy genes have been proposed to develop specific detection methods for *Colletotrichum* spp. [27]. In the present study, authors decided to focus their attention on the IGS region due its multicopy nature and high variability. The IGS region is highly polymorphic, and thus is a useful region for specific molecular diagnostic assays in fungal plant pathogens. These included *Botrytis cinerea* [28,29], *Fusarium circinatum* [30], *F. oxysporum* f. sp. *vasinfectum* [31], *Fusarium oxysporum* f. sp. *cubense* [32], *Phytophthora medicaginis* [33], *Verticillium dahliae* and *V. alboatrum* [34,35], and *Macrophomina phaseolina* [36].

Infected lupin seeds are the primary source of inoculum of *C. lupini*, and the development of a robust and sensitive diagnostic assay for early detection of contaminated seed lots able to screen before planting remains one of the most effective management strategies. The aim of this work is to provide a specific, sensitive, and simple molecular diagnostic assay for the early detection of *C. lupini* on seeds that could reduce the impact of anthracnose on lupin production.

## 2. Results

### 2.1. Evaluation of the Mycoflora Associated with Lupin Seeds

The most common fungi isolated from the three lupin species were *Alternaria alternata, Aspergillus* spp., *Cladosporium oxysporum*, *Mucor* sp., and *Penicillium* spp. Overall, fungi of 16 genera and 10 species were recovered from lupin seeds, in addition to one yeast and one sterile mycelium, as shown in Table 1. Fungal colonies developing around the seeds were transferred onto fresh Potato Dextrose Agar (PDA) plates amended with streptomycin before identification, and identification was based on morphological characteristics observed under stereoscopic and optical microscope. Fungal isolates with ambiguous taxonomic designation were analysed by molecular characterisation with the DNA regions of ITS rDNA, β-tubulin, or EF1-α.

*Alternaria alternata*, *Cladosporium oxysporum*, and *Penicillium* spp. were the main fungi in *L. albus* cv. Multitalia. *Aspergillus* spp., *Cladosporium oxysporum*, and *Penicillium* spp. were, instead, the main fungi isolated from seeds of *L. angustifolius* cv. Tango and *L. luteus* cv. Mister. Additional common fungal pathogens were isolated from the seeds such as *Fusarium tricinctum*, *F. incarnatum*, *Diaporthe* sp., *Alternaria alternata*, *Cladosporium oxysporum*, and *Botrytis cinerea*. However, such fungal strains are not known to be key pathogens of lupins. *L. albus* cv. Multitalia showed the greatest variability of fungal genera isolated (15/21), while *L. luteus* cv. Mister and *L. angustifolius* cv. Tango showed 6/21 and 9/21 fungal genera isolated, respectively (including 1 sterile mycelium). *C. lupini* was never isolated from the seeds of the three lupin species analysed, and therefore the same batch of seeds was used in the artificial inoculation experiments.

### 2.2. Artificial Inoculation of Lupin Seeds by the Water-Restriction Technique

**Figure 1:**
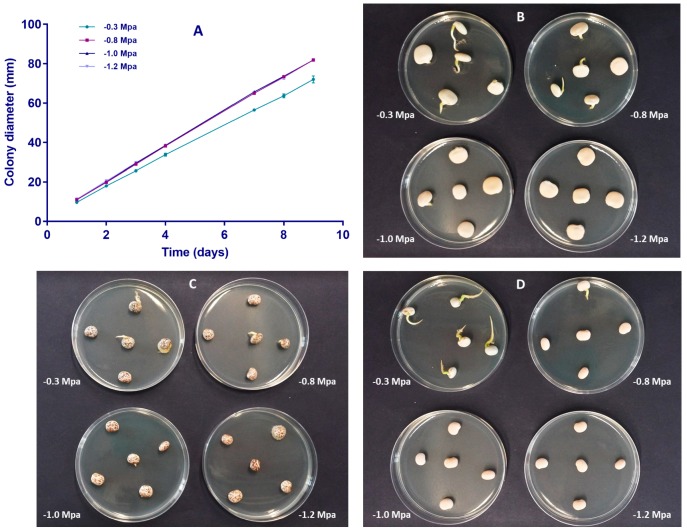
Mycelial growth of the isolate IMI504893 of *C. lupini* on Potato Dextrose Agar (PDA) osmotically modified with mannitol after 72 h of incubation (**A**). Lupin seed germination on PDA osmotically modified with mannitol: (**B**) = *L. albus* cv. Multitalia; (**C**) = *L. luteus* cv. Mister; (**D**) = *L. angustifolius* cv. Tango. −0.3 Mpa = PDA alone; −0.8 Mpa = PDA amended with 33.10 g L^−1^ mannitol; −1.0 MPa = PDA amended with 47.75 g L^−1^ mannitol; and −1.2 MPa = PDA amended with 62.46 g L^−1^ mannitol.

The influence of substrate osmotic potential on the growth rate of *C. lupini* isolate IMI504893 and on seeds germination of three lupin species was investigated (Figure 1). *C. lupini* isolate IMI504893 was growing faster on substrate characterized by osmotic potentials lower than −0.3 MPa, corresponding to the osmotic potential of PDA without mannitol (Figure 1A). No significant differences were found on colony diameter at each time on PDA osmotically modified with mannitol (−0.8, −1.0, and −1.2 MPa). On the contrary, data on non-amended PDA (−0.3 MPa) were significantly different from corresponding data on PDA osmotically modified with mannitol (Figure 1). Sterilized seeds of lupin species were placed onto PDA medium at different osmotic potentials with the aim to evaluate the influence of the substrate on seed germination (Figure 1B–D). Seed germination was strongly reduced at all the osmotic potentials used; however, after 96 h of incubation on PDA with an osmotic potential of −1.2 MPa, seeds of *L. albus* cv. Multitalia (6.67%) and of *L. luteus* cv. Mister (33.33%) started to germinate (Figure S1).

Given that the mycelial growth was stimulated at the osmotic potential of −1.2 MPa, and seed germination was inhibited at the same potential up to 72 h, the artificial inoculation of lupin seeds on PDA was adjusted at −1.2 MPa for 72 h. Inoculation was confirmed by plating 20 seeds of each cultivars on PDA amended with streptomycin. After 3 days, mycelium of *C. lupini* IMI504893 developed from all tested seeds.

### 2.3. Development of a Specific PCR Assay for C. lupini

Total genomic DNA was successfully extracted from all the fungi listed in Table 1 and Table 2. PCR amplification of the ITS region with the primer pair ITS5/ITS4 was performed on non-target species of *Colletotrichum* spp. (Table 2) and on fungi isolated from the seeds (Table 1). The ITS region was amplified as positive control for the extracted DNA, thereby excluding false negative results. Amplicons were obtained from all the tested fungi and varied between 550 and 700 bp.

The species specific primer pair CLF (5′-CCCGAGAAGGCTCCAAGTA-3′)/CLR (5′-CATAAACGCCTAAGAACCGC-3′) (Figure S2) was designed based on the multiple alignment of the IGS sequences of *C. lupini* (IMI504893, CBS109225), *C*. *tamarilloi* (CBS129955), *C*. *nymphaeae* (IMI 504889), *C*. *costaricense* (CBS211.78), *C*. *abscissum* (IMI504790), and *C*. *simmondsii* (CBS 122122). The species are taxonomically included in the *C. acutatum* species complex clades 1 and 2. The primers were designed from 100% sequence homology region of *C. lupini* isolates and from regions of the greatest sequence dissimilarity among other species.

The primer pair CLF/CLR was used in the PCR amplification with the DNA template of *C. lupini* and the *Colletotrichum* species indicated in Table 2, in addition to the DNA of the isolates of non-target species associated with lupin seeds (Table 1). Sensitivity of the PCR protocol using the primer pair CLF/CLR was performed using 10-fold dilutions ranging between 10 ng and 100 fg of mycelial DNA of *C. lupini* isolate 70555 (Figure S3). 

**Figure 2:**
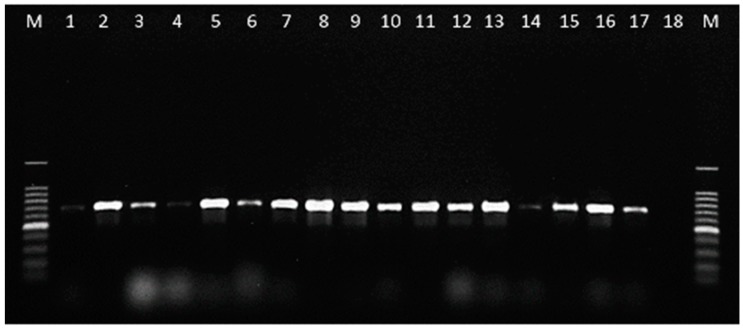
Specificity of PCR using the primer pair CLF (5′-CCCGAGAAGGCTCCAAGTA-3′)/CLR (5′-CATAAACGCCTAAGAACCGC-3′) on 17 isolates of *C. lupini* (Lane 1–17). Lane 18 = negative control (without DNA), Lane M = 100 bp DNA ladder.

PCR assay for *C. lupini* was optimized to increase primer specificity, avoiding primer mismatches and the probability of producing spurious bands or false positives. The reactions generated amplicons of the expected size of 700–750 bp in all the tested *C. lupini* isolates (Figure 2). 

**Figure 3:**
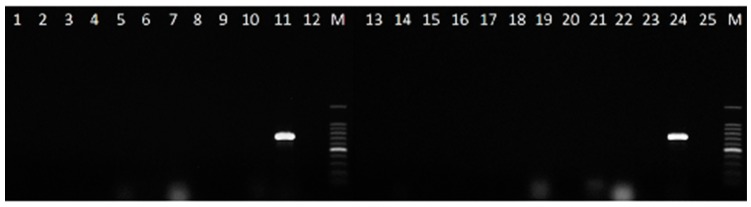
Specificity of PCR using the primer pair CLF/CLR for the detection of *C. lupini*. Lane 1 = *Alternaria alternata*, Lane 2 = *Alternaria infectoria*, Lane 3 = *Alternaria tenuissima*, Lane 4 = *Arthrinium phaeospermum*, Lane 5 = *Aspergillus* sp., Lane 6 = *Botrytis cinerea*, Lane 7 = *Chaetomium* sp., Lane 8 = *Cladosporium oxysporum*, Lane 9—*Curvularia hawaiiensis*, Lane 10 = *Diaporthe* sp., Lane 11 and 24 = *C. lupini* IMI 504893, Lane 12 and 25 = negative control (without DNA), Lane 13 = *Eurotium* sp., Lane 14 = *Fusarium incarnatum*, Lane 15 = *Fusarium tricinctum*, Lane 16 = *Lecytophora* sp., Lane 17 = *Mucor* sp., Lane 18 = *Penicillium* sp., Lane 19 = *Pleospora* sp., Lane 20 = *Sordaria fimicola*, Lane 21 = *Trichoderma* sp., Lane 22 = *Meyerozima caribbica*, Lane 23 = Sterile mycelium, and Lane M = 100 bp DNA ladder.

**Figure 4:**
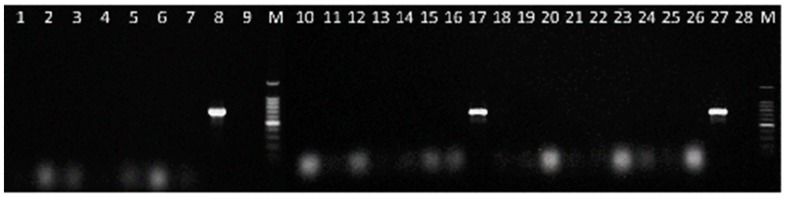
Specificity of PCR using the primer pair CLF/CLR for the detection of *C. lupini*. Lane 1 = *C. tamarilloi*, Lane 2 = *Colletotrichum* sp., Lane 3 = *C. costaricense*, Lane 4 = *C. abscissum*, Lane 5 = *C. cuscutae*, Lane 6 = *C. paranaense*, Lane 7 = *C. melonis*, Lane 8, 17 and 27 = *C. lupini* IMI 504893, Lane 9 and 28 = Negative control (without DNA), Lane 10 = *C. simmondsii*, Lane 11 = *C. nymphaeae*, Lane 12 = *C. fioriniae*, Lane 13 = *C. acutatum*, Lane 14 = *C. godetiae*, Lane 15 = *C. salicis*, Lane 16 = *C. phormii*, Lane 18 = *C. fructicola*, Lane 19 = *C. tofieldiae*, Lane 20 = *C. orchidophilum*, Lane 21 = *C. higginsianum*, Lane 22 = *C. graminicola*, Lane 23 = *C. coccodes*, Lane 24 = *C. spinaciae*, Lane 25 = *C. trichellum*, Lane 26 = *C. truncatum*, and Lane M = 100 bp DNA ladder.

The amplicons were sequenced in both directions and aligned to compare with the sequences used to design the primer pair, and to ensure the availability of the partial IGS region in all the isolates tested. Additional tests were carried out to confirm the specificity of the developed PCR assay by analysing the *Colletotrichum* species of Table 2 and the fungal strains isolated from lupin seeds of Table 1. The primer pair CLF/CLR proved specific to *C. lupini*, as no bands were obtained on all the non-target fungi tested (Figure 3 and Figure 4).

### 2.4. Detection of C. lupini in Artificially Infected Seed Samples

**Figure 5:**
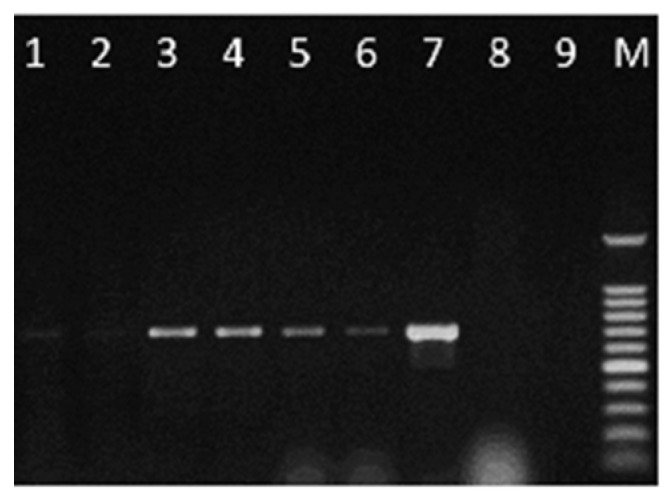
Specificity of PCR using the primer pair CLF/CLR for the detection of *C. lupini* in seed samples artificially infected (1:10,000). Lane 1–2 = *L. albus* cv. Multitalia, Lane 3–4 = *L. luteus* cv. Mister, Lane 5–6 = *L. angustifolius* cv. Tango, Lane 7 = Positive control (*C. lupini* IMI 504893), Lane 8 = Seed control (100 seeds of *L. angustifolius* cv. Tango), Lane 9 = Negative control (without DNA), and Lane M = 100 bp DNA ladder.

The species-specific primer pair CLF/CLR confirmed the presence of *C. lupini* in all infected seed lots of *L. albus* cv. Multitalia, *L. luteus* cv. Mister, and *L. angustifolius* cv. Tango, even at contamination levels as low as 0.01% (Figure 5 and Figure S4; DNA concentrations and absorbance ratios are reported in Table S1). 

The results were supported by the presence of a signal within the positive control (DNA of pure culture of *C. lupini* IMI504893), and no-signal within the negative control (not infected seed lots). The amplicons obtained from the 0.01% infection level of each species were sequenced (Table 2), with alignments carried out against the reference sequence of *C. lupini* IMI504893, showing 100% identity.

## 3. Discussion

Seed-borne fungi are one of the major biotic constraints in seed production worldwide. They are responsible for both pre- and post-emergence death of seeds reducing germination, seedling health, and plant morphology. Seed health tests include [37] visual examination and incubation methods, which require mycological skills; they are time consuming and their sensitivity/specificity is low or moderate [38,39].

In this work, a new protocol for early detection of *C. lupini* was developed based on a biological enrichment step, followed by a targeted PCR. The diagnostic assay included incubation of the seeds with amended Yeast Malt Broth to increase *C. lupini* biomass before DNA extraction. The seeds are incubated in the liquid culture, allowing the development of all culturable fungi, without the utilization of selective or semi selective media (BIO-PCR) that require specific knowledge of the target organism [38]. The biological enrichment step allows for the detection only of viable cells, and reduces false positives, which constitute a limitation of PCR diagnostic assays [40]. It also allows for the development of all the fungi present on/in the seeds and promotes the development of the target fungus biomass before DNA extraction and amplification by PCR. This step is usually very useful in cases of low infection levels. The incubation of seeds in liquid fungal growth media has been previously undertaken with positive results for the detection of *Fusarium circinatum* in pine seeds [41], *Alternaria brassicae* in cruciferous seeds [42], *Ascochyta rabiei* in chickpea seeds [43], and *Magnaporthe grisea* in rice seeds [44]. The presence of bacteria may, however, reduce fungal growth though the addition of antibiotics to the media or the use of specific media, which can circumvent these problems [43,45].

The optimal osmotic potential and the incubation period were defined according to the germination process of the seeds and the growth of *C. lupini* at different osmotic potentials. No seeds of the three lupin species germinated on PDA adjusted at −1.2 MPa at 72 h and, in the same conditions, *C. lupini* growth was stimulated. For these reasons, it was decided to perform the seed inoculation on PDA adjusted with mannitol at −1.2 MPa for 72 h. Concerning the sensibility of the method, the PCR assay developed in this study was able to reveal infection levels below 0.01% (1:10,000) in the samples tested. Assuming a 100% DNA recovery from 1 infected seed (DNA resuspended in 100 μL), 1 μL of template DNA would contain the equivalent of 0.01 infected seed in a reaction. 

The new CLF/CLR primer pair was used for amplification and sequencing of a target of about 736 bp in *C. lupini* of the IGS region of the ribosomal cluster. Bioinformatic analyses were performed on the entire sequence of the clusters, and more specifically the complete IGS region of species belonging to the *Colletotrichum acutatum* species complex clades 1 and 2 (*C. lupini*, *C. tamarilloi*, *C. nymphaeae*, *C. costaricense*, *C. abscissum*, and *C. simmondsii*). PCR-based detection systems exhibit higher levels of sensitivity than conventional techniques. However, when applied to seed tests they require the isolation of high-quality DNA of target organisms for detection above the background noise of other organisms and inhibitory seed-derived compounds. Niepold (2003) designed a primer pair from an already sequenced ITS1 region in order to detect *C. lupini*. The sequences, when subjected to a BLASTn search, showed 100% identity with many other *Colletotrichum* species (e.g., *C. acutatum*, *C. scovillei*, *C. simmondsii*, *C. salicis*, *C. godetiae*, *C. phormii*, and *C. nymphaeae*). More recently, Szuszkiewicz (2016), during a study on the molecular detection of *C. lupini* in lupin seeds, developed a species-specific primer pair based on ITS region that was specific to the *C. acutatum* species complex. Dubrulle et al. (2019) used specific primers targeting a single copy gene to detect *C. lupini* DNA in winter and spring lupin fields in France. Of the 47 samples collected, *C. lupini* was quantified in six samples above the limit of quantification (LOQ) (10^−3^ ng DNA), although all at a very low level (between 10^−3^ and 5 × 10^−3^ ng DNA) [24]. IGS region could be an attractive alternative to the ITS region when closely related taxa or even different species need to be investigated. The IGS region tends to accumulate more variability than the ITS region of the ribosomal cluster and, therefore, contains more sequence polymorphisms. It occurs in multiple copies, increasing the sensitivity of PCR-based diagnostics compared to single-copy target sequences and allowing its amplification using generic PCR primers [46]. The greatest amount of sequence variation in the ribosomal cluster exists within the IGS region, even if it poses more difficulties for amplification and sequencing, as it is long (2000–5000 bp) and rich in GC [47]. To our knowledge, besides the potential of the target, no published studies reported the use of the IGS region for the molecular diagnosis of *Colletotrichum*. 

Seed-borne mycoflora of the three lupin species used in this study *L. albus* cv. Multitalia, *L. luteus* cv. Mister, and *L. angustifolius* cv. Tango were evaluated for three main reasons: (i) to assess the level of seed contamination/infection by cultivable fungi, (ii) to collect a data set of fungi associated with lupin seeds to be used as non-target fungi in the development of the species specific PCR assay, and (iii) to investigate the presence of *C. lupini* in the seed lots used. *L. albus*, *L. luteus*, and *L. angustifolius* showed 84, 82, and 60% seed contamination, respectively. Most of the fungi recovered on seeds of the three lupin species were also reported in several published studies on the mycoflora associated with white, yellow, and blue lupin [48,49,50,51,52].

In this study, *C. lupini* was never isolated from seeds of the three lupin species analysed, and therefore seeds were used in the artificial inoculation experiments and in the preparation of seed batches by mixing artificially infected seeds and healthy seeds in different proportions. Seeds were kept in direct contact with developing colonies of the fungal pathogen on agar media amended with an osmotic compound that inhibited seed germination. 2,4-D (sodium salt of 2,4-dichlorophenoxyacetic acid), mannitol, sodium, and potassium chlorides can be used to modify the osmotic potential of PDA [53]. In this study, mannitol was used to obtain PDA with different osmotic potentials. Mycelial growth of *C. lupini* was stimulated at all the osmotic potentials higher than −0.3 MPa (PDA without mannitol). In general, development of fungi is not reduced by osmotic potential lower than −2.0 MPa, and in the range between −0.3 and −1.0 MPa growth of some fungi are stimulated. Similar results were reported for the inoculation of *Phaseolus vulgaris* seeds with *Colletotrichum lindemutianum* and cotton seeds with *Colletotrichum gossypii* var. *cephalosporioides* [54,55]. Germination rate of each lupin cultivar used in this study decreased as osmotic potential increased. Similar results were observed in cotton, rice, sunflower, and common bean seeds [53,56,57]. Several studies reported that the incubation period to obtain inoculated seeds ranged between 24 and 144 h [58,59,60,61]. 

DNA concentration has been taken into consideration by Cullen et al. [62] to exploit the sensitivity of PCR for the specific detection of *Helminthosporium solani* in seeded soils. The molecular detection of fungi on seeds can be influenced by factors such as low concentration of target pathogen, the presence of other organisms, and other natural compounds of the seeds. Gondran and Pacault [17] recommended that seed testing for *L. albus* should detect as little as one anthracnose-infected lupin seed in 10,000 (0.01%). Shea et al. [63] reported that in Western Australia, a PCR test, based on research carried out at the Centre for Legumes in Mediterranean Agriculture and the State Agricultural Biotechnology Centre, was able to detect one infected seed in 10,000. The test was improved over time to become quantitative, providing an estimation of the seed infection level based on the amount of *C. lupini* DNA present in the sample. However, the PCR protocol has not been published but only described as a useful seed-testing service for lupin growers in high-risk areas. 

The protocol described in this work is a useful diagnostic tool for the routine detection of *C. lupini* in seed lots: the assay is simple, reliable, economic, and sensitive. Over time, with a greater understanding of the anthracnose disease, seed testing should be widely utilised as a tool for seed certification, for local seed supply, and for international seed biosecurity. The use of pathogen-free seeds is an essential strategy for the integrated and sustainable management of plant diseases.

## 4. Materials and Methods

### 4.1. Fungal Cultures

Isolates used in this study and relative information are summarized in Table 2. Purified fungal cultures were routinely grown on Potato Dextrose Agar (PDA, Difco Laboratories, Detroit, MI, USA) and were maintained on PDA slants covered with mineral oil at 4 °C for long-term storage. 

### 4.2. DNA Extraction

Fungal mycelium for DNA extraction was grown in 125 mL of Yeast Malt Broth (YMB—0.3% yeast extract, 0.3% mal extract, 0.5% peptone, and 1% dextrose) at 150 rpm for 2–4 days at 24 ± 1 °C in the dark. Mycelium was harvested by filtration through sterile Miracloth (Calbiochem, San Diego, CA, USA), washed thoroughly using sterile distilled water, and pressed dry between sterile paper towels. The harvested mycelium was either used immediately for DNA extraction or stored at −20 °C until use. 

Total genomic DNA was extracted using the SDS-CTAB method described by Kim et al. [63] with some modifications. Mycelium (200 mg) was placed into a 2 mL extraction tube prefilled with 0.5 mm Silica glass beads (acid washed) (Benchmarck Scientific Inc., Sayreville, NJ, USA), 50 mg of PVP40 (Sigma-Aldrich, Saint Louis, MS, USA), and 400 µL of ice-cold lysis buffer (150 mM NaCl, 50 mM EDTA, 10 mM Tris-HCl pH 7.4, 30 µg mL^−1^ Proteinase K). The mycelium was homogenized by a bead-beating method using the BeadBug™ Microtube homogenizer (Benchmarck Scientific Inc., Sayreville, NJ, USA). Tubes were subjected to three beating cycles of 30 s at 4000 rpm followed by a 30 s interval. During the interval and after the cycle, the samples were cooled down in ice. SDS was added to a final concentration of 2% (w/v), and the mixture was incubated at 65 °C for 40 min. The lysed suspension was centrifuged for 10 min at 4 °C and 2500 g, the volume of supernatant was measured, and the NaCl concentration was then adjusted to 1.4 M and 1/10 volume of 10% CTAB buffer (10% CTAB, 500 mM Tris-HC1, 100 mM EDTA, and pH 8.0) and added. After thorough mixing, the solution was incubated at 65 °C for 10 min. After cooling at 15 °C for 2 min, extraction with chloroform-isoamyl alcohol (24:1 v/v) was conducted for 10 min at 4 °C and 6700 *g*. DNA was precipitated with two volumes of 95% cold ethanol. Samples were stored at −20 °C (for a minimum of 1 h) and centrifuged for 1 min at 4 °C and 11,600 g, and the resulting pellet was rinsed once with 70% cold ethanol, vacuum-dried, and dissolved in sterile nuclease-free water. DNA solutions were stored at −20 °C until use.

DNA concentration was estimated with a GeneQuant II spectrophotometer (Pharmacia Biotech, Cambridge, UK), whereas its integrity was examined visually by gel electrophoresis on 0.8% (w/v) agarose gels run in 0.5× TBE buffer followed by GelRed™ staining (Biotium Inc., Fremont, CA, USA), according to the manufacturer’s instructions. Following quantification, the genomic DNA was diluted to a final concentration of 25–50 ng µL^−1^.

### 4.3. Evaluation of Mycoflora Associated with Lupin Seeds

A hundred non-sterilized seeds of *Lupinus albus* cv. Multitalia, *L. luteus* cv. Mister, and *L. angustifolius* cv. Tango were plated on PDA amended with streptomycin (300 mg L^−1^), five seeds per Petri plate, and incubated at 24 ± 1 °C under a 12 h near-ultraviolet light/12 light cycle for 7 days. 

Plates were examined under stereoscopic and compound microscopes to identify the developing fungal colonies. Hyphal-tip and/or single-spore isolation techniques were used to obtain pure cultures. Fungi were identified according to their morphological properties and, when necessary, by DNA sequencing.

The ITS and 5.8 S region of rDNA was amplified with the primer pair ITS5 (5′-GGAAGTAAAAGTCGTAACAAGG-3′), and ITS4 (5′-TCCTCCGCTTATTGATATGC-3′) [25]. The partial β-tubulin gene region was amplified with the primer pair BT2A (5′-GGTAACCAAATCGGTGCTGCTTTC-3′) and BT2B (5′ACCCTCAGTGTAGTGACCCTTGGC-3′) [64]. The partial sequences of the elongation factor-1 alpha gene (*tef1*) were amplified with the primer pair EF1-728F (5′-CATCGAGAAGTTCGAGAAGG-3′) and EF1-986R (5′-TACTTGAAGGAACCCTTACC-3′) [65]. Sequence chromatograms obtained by direct sequencing were visualised and analysed using Chromas Lite (v. 2.1.1) and BioEdit (v. 7.2.5) programs.

Each sequence was used to perform individual nucleotide-nucleotide searches with the BLASTn algorithm at the NCBI website (https://blast.ncbi.nlm.nih.gov/). Sequence-based identities with a cutoff of 97% or greater were considered significant in this study, and the best hit was defined as the sequence with the highest maximum identity to the query sequence.

Seed germination was determined according to the procedure described by the International Seed Testing Association (ISTA, 2019).

### 4.4. Genome Data and Analysis of Ribosomal IGS Region

Based on whole genome sequencing project available on GenBank, a set of four raw sequencing data information sets from high-throughput sequencing platforms stored in the sequences reads archive (SRA) database were downloaded: accession number for *C. simmondsii* SRP074810, for *C. nymphaeae* SRP074816, for *C. fioriniae* SRP074685, and for *C. salicis* SRP074780 [66]. Raw data reads were assembled using SPAdes v 3.11.1 [67], and high-coverage scaffolds were extracted with a homemade script. Scaffolds corresponding to the ribosomal RNA encoding gene cluster were identified by BLASTN searches based on available references. When the software failed to assemble the region in one unique scaffold, a manual approach based on local alignment of raw reads to assembled scaffolds with Bowtie2 [68] was used to increase the size of the scaffolds and to reach an overlap of at least 50 bp.

Annotated genome sequences of *C. lupini* IMI504884 and CBS109225, *C. abscissum* IMI504890, *C. costaricense* IMI309622, and *C. tamarilloi* CBS129955 were downloaded from the MycoCosm portal [69] and the ribosomal cluster sequence retrieved.

All sequences were analysed and compared. The sequences starting with the forward primer CNL12 (5′CTGAACGCCTCTAAGTCAG3′) and ending with the reverse primer CNS1 (5′GAGACAAGCATATGACTACTG3′), which are universal primers, located at the 3′ end of the 28S gene and the 5′ end of the 18S gene, respectively, were used for the amplification of complete IGS region in many fungi [25,70].

The sequences were aligned using the MAFFT v. 7 [71] and were visually checked for regions having homologies among isolates of *C. lupini* but not among other *C. acutatum* species.

### 4.5. Development of Specific Oligonucleotide PCR Primers

Conserved regions among the isolates that were specific to *C. lupini* were selected to design species-specific oligonucleotides. Two primers, forward and reverse, were designed using Primer3Plus online software with default options [72]. The forward (CLF: 5′CCCGAGAAGGCTCCAAGTA3′) and reverse (CLR: 5′CATAAACGCCTAAGAACCGC3′) primers yielded a product of 736 bp. The theoretical specificity of the primer set was checked with the sequences from the other fungi in the GenBank by using BLASTn analysis.

### 4.6. Development of a Specific PCR Assay for C. lupini

The specific primer pair CLF/CLR designed during this study was used for amplification. The optimized PCR amplification protocol was performed in a total volume of 25 µL containing 25–50 ng of template DNA, 0.2 µM of each oligonucleotide primer, and 12.5 μL of GoTaq^®^ Green Master Mix (Promega, Madison, WI, USA). An initial denaturation step of 94 °C for 2 min was followed by 25 cycles of a 45 s at 94 °C (denaturation step), 15 s at 63 °C (annealing step), and 45 s at 72 °C (extension step). After 25 cycles, samples were incubated for 7 min at 72 °C (final extension step). Negative controls (no DNA) were included for each set of reactions.

Templates were represented by (i) DNA of *C. lupini* and other *Colletotrichum* species (Table 2) and (ii) DNA of isolates of non-target fungi associated with lupin seeds and isolated in this work (Table 1). When more than one species was isolated (e.g., *Aspergillus* spp.), the most representative was chosen. PCR products were analysed by electrophoresis in 0.5× TBE buffer with 2% (w/v) agarose gels and detected by UV fluorescence after GelRed™ staining (Biotium Inc., Fremont, CA, USA), according to the manufacturer’s instructions. The 100 bp DNA ladder (Promega, Madison, WI, USA) was used as molecular size marker. PCR products of 17 *C. lupini* isolates were purified using the QIAquick PCR purification Kit (Qiagen, Milano, Italy) and sequenced in both directions to confirm the nucleotide sequence. Sequence chromatograms were visualised and edited using Chromas Lite (v. 2.1.1) and BioEdit v.7.2.5 [73] and deposited in GenBank (Table 2).

### 4.7. Artificial Inoculation of Lupin Seeds by Water Restriction Technique

The water restriction technique [56], also known as ‘osmo-priming’ process [74] is based on the control of the osmotic potential of the substrate to inhibit or reduce the seed germination process, which allows for the inoculation of seeds without producing any radicle protrusion during the incubation period. This technique, at the same time, does not affect microorganisms associated with seeds.

Before the artificial inoculations of lupin seeds, in order to choose the best conditions, we evaluated the effect of water restriction on the mycelial growth of the pathogen and on lupin seed germination. The effect of water restriction on *C. lupini*, and mycelial growth on osmotically modified PDA medium containing mannitol, was evaluated. Osmotic potential of −0.8 (33.10 g L^−1^), −1.0 (47.75 g L^−1^), and −1.2 (62.46 g L^−1^) MPa was used for the incorporation of the osmotic solute into the PDA medium. The osmotic potential of PDA without the solute was estimated to be −0.35 MPa [57]. Agar plugs (6 mm diameter) were removed from the edge of an actively growing colony of *C. lupini* (isolate IMI504893) and placed at the center of PDA plates (9 cm) amended with mannitol. Plates were incubated at 24 ± 1 °C under a 12 h near-ultraviolet light/12 light cycle for 9 days. Mycelial growth was measured taking the average of two diameters of colonies at appropriate angles to each other at different day intervals. The experiment was performed in triplicate.

Two-way analysis of variance (ANOVA) and Tukey’s multiple-comparison post-test were conducted with colony diameter data obtained from the experiment using GraphPad Prism version 7.00 for Windows (GraphPad Software, La Jolla, CA, USA). Differences were considered significant at a *p* value < 0.05.

To evaluate the effect of water restriction in the absence of the fungus, seeds of the three lupin species were placed on Petri plates containing PDA osmotically modified with mannitol for 7 days, as described above. Seeds of *L. albus* cv. Multitalia, *L. luteus* cv. Mister, and *L. angustifolius* cv. Tango were disinfected with 1% NaOCl (v/v) for 2 min, rinsed twice in sterile distilled water, and dried at room temperature on sterile filter paper before plating. Percentage of germinated seeds was recorded.

At the end of these experiments, according to the results obtained, lupin seeds were inoculated with the isolate IMI504893 of *C. lupini*, using the ‘osmo-priming’ technique with the following conditions: osmotic potential adjusted to −1.2 MPa with mannitol over a 72 h incubation period. Lupin seeds of each species were disinfected and placed in a single layer on the fungal colonies under the same conditions previously described. The seeds were then shade-dried and kept at 4 °C for future mixing with healthy seeds. Inoculation accuracy was confirmed at the end of the experiments, and after six months by placing 20 seeds on the surface of PDA plates amended with streptomycin (300 mg L^−1^) for 1 week at 24 ± 1 °C.

### 4.8. Detection of C. lupini in Seed Samples with Different Fungal Incidence

To determine the efficacy and the sensibility of the PCR technique in the detection of *C. lupini* in lupin seed samples, seed batches were prepared by mixing infected seeds and healthy seeds in the following proportions to produce seed lots with the following infection levels: 1:10 (10%); 1:100 (1%); 1:1000 (0.1%); 1:10,000 (0.01%), and 0.00%. One thousand seed batches’ weights were 281.42 g for *L. albus* cv. Multitalia, 135.46 g for *L. luteus* cv. Mister, and 146.85 g for *L. angustifolius* cv. Tango. The artificially infected seeds, before mixing, were disinfected with 1% NaOCl (v/v) for 2 min, rinsed twice in sterile distilled water, and dried at room temperature on sterile filter paper.

Seed batches were placed in sterile Erlenmeyer flasks (between 250 and 6000 mL depending on the cultivar and the batch), covered with liquid culture YMB medium amended with streptomycin (0.03%), ampicillin (0.0025%) and lactic acid (0.1%), and incubated for 72 h at 24 ± 1 °C with continuous shaking (150 rev min^−1^). This incubation step is an enrichment phase that allows an optimal increase of the fungal biomass from seed.

At the end of the incubation period, the content of each flask was harvested by filtration through sterile Miracloth (Calbiochem, San Diego, CA, USA). The seeds were discarded after harvesting the mycelium. After thoroughly washing with deionized sterile water, the resulting mycelium was pressed dry between sterile paper towels and was either used immediately for DNA extraction or stored at −20 °C until use. 

DNA extraction, PCR, and agarose electrophoresis were undertaken as described above. The universal primer pair ITS5/ITS4 [25] was used as a positive control to assess the quality of the extracted DNA. DNA from pure cultures of *C. lupini* isolate IMI504893 was used as a positive control in the assays. Furthermore, the specific PCR product obtained was eluted by gel extraction, and DNA sequencing was carried out to confirm that the designed primers correctly amplified the expected consensus region of the target organism. The molecular assay was carried out with two replicates, and the experiments were repeated twice for each level of seed infection.

## Figures and Tables

**Table 1:** Percentage of contaminated seeds and mycoflora composition of the studied lupin varieties. Methods and accession number of genetic loci used for the characterization of main contaminants are also reported.

*Lupinus* spp.	*albus*	*luteus*	*angustifolius*	Method ^1^	Loci Used for Molecular Characterization		
cultivar	Multitalia	Mister	Tango				
Contamination (%)	84	82	60		Accession of Reference Sequences		
Germination (%)	85.5	70.5	93.5		ITS	TUB	TEF
*Alternaria alternata*	42	4	2	M&G	MK560162	MK567923	-
*Alternaria infectoria*	2	-	-	M&G	MK560163	MK567924	-
*Alternaria tenuissima*	4	-	-	M&G	MK560164	MK567925	-
*Arthrinium phaeospermum*	2	-	-	M&G	MK560165	-	-
*Aspergillus* spp.	6	40	10	M	-	-	-
*Botrytis cinerea*	2	-	-	M&G	MK560166	-	-
*Chaetomium* sp.	-	-	2	M	-	-	-
*Cladosporium oxysporum*	14	28	22	M&G	MK560167	MK567926	-
*Curvularia hawaiiensis*	2	-	-	M&G	MK560168	MK567927	-
*Diaporthe* sp.	2	-	-	M	-	-	-
*Eurotium* sp.	-	-	2	M&G	MK560169	-	-
*Fusarium incarnatum*	2	-	-	M	-	-	MK567921
*Fusarium tricinctum*	2	-	-	M	-	-	MK567922
*Lecythophora* sp.	-	-	2	M&G	MK560170	MK567928	-
*Mucor* sp.	8	8	-	M	-	-	-
*Penicillium* spp.	12	22	22	M	-	-	-
*Sordaria fimicola*	-	2	-	M&G	MK560171	MK567929	-
*Trichoderma* spp.	2	-	2	M	-	-	-
*Meyerozima caribbica*	2	-	-	M&G	MK560172	-	-
Sterile mycelium	-	-	2	M	-	-	-

^1^ Method used for the characterization: M indicates the strains were characterized morphologically, and M&G indicates morphologically and genetically.

**Table 2:** *Colletotrichum* isolates used in this study and relative information.

*Colletotrichum* spp.	Isolate ^1^	Host	Country ^2^	GenBank Accession Numbers ^3^		
				ITS	rDNA cluster	IGS
*C. lupini*	PT30, RB020	*Lupinus albus*	PT	MK463722	-	**MK567906**
*C. lupini*	CBS129944, RB042	*Cinnamomum* sp.	PT	MH865693	-	**MK567907**
*C. lupini*	CSL1294, RB116	*Lupinus polyphyllus*	GB	MK463723	-	**MK567908**
*C. lupini*	G52, RB119	*Lupinus albus*	DE	MK463724	-	**MK567909**
*C. lupini*	96A649, RB120	*Lupinus polyphyllus*	AU	MK463725	-	**MK567910**
*C. lupini*	IMI504884, RB121	*Lupinus albus*	CA	KJ018635	-	**MK567911**
*C. lupini*	C3, RB122	*Lupinus luteus*	PL	MK463726	-	**MK567912**
*C. lupini*	IMI504885, RB123	*Lupinus albus*	ZA	MK463727	-	**MK567913**
*C. lupini*	70555, RB124	*Lupinus albus*	CL	MK463728	-	**MK567914**
*C. lupini*	CBS109224, RB125	*Lupinus albus*	AT	JQ948172	-	**MK567915**
*C. lupini*	PT702, RB127	*Olea europea*	ES	MK463729	-	**MK567916**
*C. lupini*	IMI350308, RB147	*Lupinus* sp.	GB	MK463730	-	**MK567917**
*C. lupini*	CBS109221, RB172	*Lupinus albus*	DE	JQ948169	-	**MK567918**
*C. lupini*	CBS109225, RB173	*Lupinus albus*	US	JQ948155	**MK541036**	-
*C. lupini*	IMI375715, RB217	*Lupinus albus*	AU	JQ948161	-	**MK567919**
*C. lupini*	IMI504893, RB221	*Lupinus* sp.	FR	MK463733	**MK541037**	-
*C. lupini*	CBS509.97, RB235	*Lupinus albus*	FR	JQ948159	-	**MK567920**
*C. abscissum*	IMI504790, RB197	*Citrus x sinensis*	US	KT153558	**MK541030**	-
*C. costaricense*	CBS211.78, RB184	*Coffea* sp.	CR	JQ948181	**MK541033**	-
*C. cuscutae*	IMI304802, RB216	*Cuscuta* sp.	DM	JQ948195	-	-
*C. melonis*	CBS134730, RB237	*Malus domestica*	BR	KC204997	-	-
*C. paranaense*	IMI384185, RB218	*Caryocar brasiliense*	BR	JQ948191	-	-
*C. tamarilloi*	CBS129955, RB018	*Solanum betaceum*	CO	JQ948189	**MK541029**	-
*Colletotrichum* sp.	CBS101611, RB170	Fern	CR	JQ948196	-	-
*C. nymphaeae*	IMI 504889, RB190	*Fragaria x ananassa*	DK	KT153561	**MK541035**	-
*C. simmondsii*	CBS 122122, RB179	*Carica papaya*	AU	JQ948276	**MK541034**	-
*C. fioriniae*	IMI 504882, RB111	*Fragaria x ananassa*	NZ	KT153562	**MK541031**	-
*C. acutatum*	CBS 112980, RB175	*Pinus radiata*	ZA	JQ948356	-	-
*C. godetiae*	CBS 193.32, RB019	*Olea europaea*	IT	JQ948415	-	-
*C. phormii*	CBS 102054, RB171	*Phormium* sp.	NZ	JQ948448	-	-
*C. salicis*	CBS 607.94, RB157	*Salix* sp.	NL	JQ948460	**MK541032**	-
*C. coccodes*	RB302	*Solanum lycopersicum*	IT	**MK531998**	-	-
*C. spinaciae*	RB305	*Spinacia oleracea*	IT	**MK531997**	-	-
*C. higginsianum*	IMI 349063, RB300	*Brassica chinensis*	TT	JQ005760	-	-
*C. graminicola*	CBS130836, RB301	*Zea mays*	US	JQ005767	-	-
*C. fructicola*	CSL386, RB003	*Fragaria x ananassa*	US	KM246513	-	-
*C. orchidophilum*	IMI309357, RB209	*Phalaenopsis* sp.	GB	JQ948153	-	-
*C. tofieldiae*	IMI288810, RB164	*Dianthus* sp.	GB	GU227803	-	-
*C. trichellum*	RB306	*Hedera* sp.	IT	**MK532000**	-	-
*C. truncatum*	RB308	*Glycine max*	AR	**MK531999**	-	-

^1^ CBS: culture collection of Centraalbureau voor Schimmecultures, Fungal Biodiversity Centre, Utrecht, The Netherlands; IMI: culture collection of CABI Europe UK Centre, Egham, UK; RB: personal collection of Riccardo Baroncelli. ^2^ ISO 3166-1 alpha-2 code related to the country of origin. ^3^ in bold are highlithed the sequences produced in this study.

## Supplementary Materials

The following are available online at https://www.mdpi.com/2223-7747/8/7/222/s1. Figure S1: Influence of PDA osmotically modified with mannitol on seed germination of *L. albus* cv. Multitalia, *L. luteus* cv. Mister and *L. angustifolius* cv. Tango. −0.3 MPa = PDA alone; −0.8 MPa = PDA amended with 33.10 g L^−1^ mannitol; −1.0 MPa = PDA amended with 47.75 g L^−1^ mannitol; and −1.2 MPa = PDA amended with 62.46 g L^−1^ mannitol. The red rectangle indicates the osmotic potential and the time chosen for the artificial inoculations of lupin seeds. Figure S2: Specific 736 bp IGS region flanked by the primer pair CLR/CLF. The sequences of *C. lupini* RB221 (IMI504893) and RB173 (CBS109225) were used as references. Some isolates of *C. acutatum* species complex belonging to clades 1 and 2 were compared and used to design the specific primer pair. Figure S3: Sensitivity of PCR using the primer pair CLF/CLR for the detection of *C. lupini*. The assay was performed using 10-fold dilutions ranging between 10 ng and 100 fg of mycelial DNA of *C. lupini* isolate 70555 (Lanes 1-6). Lane 7 = Negative control (without DNA). Marker =100 bp DNA ladder. Figure S4: Specificity of PCR with the primer pair CLF/CLR for detection of *C. lupini* in seed samples artificially infected (a = 1:10, b = 1:100, c = 1:1000). Lane 1–2 = *L. albus* cv. Multitalia, Lane 3–4 = *L. luteus* cv. Mister, Lane 5–6 = *L. angustifolius* cv. Tango, Lane 7 = positive control (*C. lupini* IMI504893), Lane 8a = seed control (100 seeds of *L. albus* cv. Multitalia), Lane 8b = seed control (100 seeds of = *L. angustifolius* cv. Tango), Lane 8c = seed control (100 seeds of = *L. luteus* cv. Mister), Lane 9 = negative control (without DNA), and Lane M = 100 bp DNA ladder. Table S1: DNA concentrations and absorbance ratios of *Lupinus* spp. seed batches artificially infected with *Colletotrichum lupini* IMI504893.
