# Supplementary material for: Molecular Detection of the Seed-Borne Pathogen Colletotrichum lupini Targeting the Hyper-Variable IGS Region of the Ribosomal Cluster
Source: Plants (Basel). 2019 Jul 14;8(7):222. doi: 10.3390/plants8070222 (PMC6681257; doi:10.3390/plants8070222)
Supplement: Supplementary file 1 [file plants-08-00222-s001.zip › Figure S2.pdf]

```
                          CLF →
C.lupini RB221            CCCGAGAAGG CTCCAAGTAC CCTAGAACAG CT-------- ---------- ---CCCCAGG TACCCCAGAA CACGT-AGAC CAGGTACCCT
C.lupini RB173            CCCGAGAAGG CTCCAAGTAC CCTAGAACAG CT-------- ---------- ---CCCCAGG TACCCCAGAA CACGT-AGAC CAGGTACCCT
C.tamarilloi RB018        CCCAAGACCG CCCCAGGTAC CCTAGAGCAG ACG------- ---------- ---CCCCAGG TACCCTAGAA CACGT-AGAC CAGGTACCCT
C.nymphaeae RB190         CCCAAGACAG CCCCAGGTAC CCTAGAGCAG GTGTCGCCAG CCCCCCAAGA CCGCCCCAGG TACCCTAGAA CAGGT-GCAC CAGGTACCCT
C.costaricense RB184      CCCGAGGAGG CCCCAGGTAC CCTAGAACAG ACG------- ---------- ---CCCCAGG TACCCTAGAA CAGGT-CC-C CAGGTACCCT
C.abscissum RB197         TAGAACAGCT CACCAGGTAC CCTAGAGCAG GTGCCGCCAG CTCCCCAAGA CCGCCCCAGG TACCCTAGAG CAGGTGCTGC CAGGTACCCT
C.simmondsii RB179        TAGAGCAAGT CCCCAAGTAC CCTAGAGCAG GCACCGCAAA CACCCCAAGG CCGCCCCAGG TACCCTAGAA CAGGT-ACAC TAGGTACCCT

C.lupini RB221            AGAGCAAGCG CTATAAGGGC CCTAGGGCCG GCCTAAATAG CTTAAAGCGA AGC------- ---CCTA-GG CATTATAAAA AGCGGTCGGA
C.lupini RB173            AGAGCAAGCG CTATAAGGGC CCTAGGGCCG GCCTAAATAG CTTAAAGCGA AGC------- ---CCTA-GG TATTATAAAA AGCGGTCGGA
C.tamarilloi RB018        AGAGCAAGCG CTATAAGGGC CCTGGGGCCG GCCTAAATAG CTTAAAACGA AGC------- ---CCTA-GG TGTTATAAAA AGCGGTCGGA
C.nymphaeae RB190         AGAGCAAGCG CCACGAGGGC CCTAGGGCCG GCCTAAATAG CTTGAAACGA AGC------- ---CCTAA-G TAGTATAAAA AGCGGCCAGA
C.costaricense RB184      AGAACAGGTG CCGCGAGTGC CCCGAGAAGG CTCCAGGTAC CCTAGAGT-A AGCGCTATAA GGCCCTTAGG TATTATAAAA AGCGGTCGGA
C.abscissum RB197         AGGGCAAGCG CTATAAGGGC CCCAGGGCCG GCCCAAATAG CTTAAAACGA AGC------- ---CCTA-GG TATTATAAAA AGCGGTCGGA
C.simmondsii RB179        AGAGCAAGCG CTACGCGGGC CCTAGGGCCG GTCTAAATAG CTTAAAACGA AGC------- ---CCAAA-G TAGTATAAAA AGCGGCCAGA

C.lupini RB221            GGGCCTAGGT ACCCTAGAAG AGAACTTGGT AAGAATAAAT AAGATTATTC GGGAAATAAA AACTGAACTA CGTACTGAGT ATGAGGTAAT
C.lupini RB173            GGGCCTAGGT ACCCTAGAAG AGAACTTGGT AAGAATAAAT AAGATTATTC GGGAAATAAA AACTGAACTA CGTACTGAGT ATGAGGTAAT
C.tamarilloi RB018        GGGCCTAGGT ACCCTAGAAG AGAACTTGAT GAGAATGAAT AAGATTATTC GGGAAATAAA AACGGAACTA CGTACTGAGT ATGAGGTAAT
C.nymphaeae RB190         GGGCCTAGGT ACCCTAGAAG AGAACTTAAT AAGGATAAGT AGGATTATTC GGGAAATAAA AACTGAACTA CGTAGTAAGC ATAAGGTAAT
C.costaricense RB184      GGGCCCAGGT ACCCTAGAAA AGAACTCTCT AAGAATAAAT AAGATTATTC GGGAAATAAA AACTGAACTA CGTACTGGGT ACGAGGTGAT
C.abscissum RB197         GGGCCTAGGT ACCCTGAAAG AGAACTCGAT AAGAACAAAT AAGATTATTC GGGAAATAAA AACTGAACTA CGTACTAAGT AAGAGGGAAT
C.simmondsii RB179        GGGCCCAGGT ACCCTAAAAG AGAACTTAAT AAGGATAAGT AGGATTATTC GGGAAATAAA AACTGAACTA CGTAGTAAGT CTAGGGTAAT

C.lupini RB221            ACGTCGATTT TAACTTGGAA TGGTTGTAGA AGATAAGTAC GTTTATACCT TTCTAGGGTA CGAAAAACGG AATTTAATAA TAAAAAGCTA
C.lupini RB173            ACGTCGATTT TAACTTGGAA TGGTTGTAGA AGATAAGTAC GTTTATACCT TTCTAGGGTA CGAAAAACGG AATTTAATAA TAAAAAGCTA
C.tamarilloi RB018        ACGTCGATTT TAACTTGGAA TGGTTGTAGA AGATAAGTAC GTTTATACCT TTTTAGGGTA CGAAAAACGG AATTTAATAA TAAAAAGCCA
C.nymphaeae RB190         ATGTTGATCT TGACTTGGAG TAAGTACAAG AGATAGGTGC GTTTATACCT TTCTAGGGTA CGAAGAACGA AGTAAAATAA TAAAAAGCTG
C.costaricense RB184      GCGTTGATTT TAACCTGGGA TAGTTTTAGA AGATAAGTAC GTTTATACCT TTCTAGGGTA CGAAAAACGG AATTTAATAA TAAAAAGCTA
C.abscissum RB197         ACGTTGATTT TGACTTGGAG TAGTTTTAGA GAATAAGTAC GTTTCTACCT TTCTAGGGTA CGAAAAAGGG AATTTAATAA TAAAAAGCTA
C.simmondsii RB179        ATATTGATCT TAACTTGGAA TAAGTATAAG AGATAGGTGT GTTTATATCT TTCTAGGGTA CGAAGAACGA AATAAAATAA TAAAAAGCTA

C.lupini RB221            GGTAGAGCGT TCTTAATTAG ATAGGGGAGT TTTATTTTTT ATATATTATA GTACTACGCT TTATAAGAAC TTTATAATAC AAGAGTATGA
C.lupini RB173            GGTAGAGCGT TCTTAATTAG ATAGGGGAGT TTTATTTTTT ATATATTATA GTACTACGCT TTATAAGAAC TTTATAATAC AAGAGTATGA
C.tamarilloi RB018        GATAGAGCGT TCTTTGTTAG ATAGGGGAGT TTTATTCTAT ATATATTATA GTACTACGCT TTATAAGAAC TTGATAATAT AAAAGTATAA
C.nymphaeae RB190         GATGGAGCGT TTTATATTAG ACAGGGGAGT TTTGTTTTAT ATATACTATA ATACTACGTT TTAAAAGAAC TTTATAATAT AATAATGTGA
C.costaricense RB184      GGTAGAGCGT TCTTTATTAG ATAGGGGAGT TTTATTCTTT ATATATTATA ATACTGCGCT TTATAGGAAC TTTATAATAT AAAAGTATGA
C.abscissum RB197         GGTAGAGCGT TTTTTATTAG ATGGGGGGGG TTTATTCTCT ATATATTATA GTACTACGCT GTGTAGGAAC TTGATAATGT AAAAGTATAA
C.simmondsii RB179        GATGGAGCGT TTTATAATAG ATGGGGGAGT TTTATCCTAT ATGTATTATG GTACCACGTT TTTATAGAAG TTTGTAATGT AAAAATATAA

C.lupini RB221            AAAAAGAAGG AAAAAATAG- ------AAAA CTACTACGTA AGTTCATAAT GAAGTATACA AACAATATAT TTAGAGCTTT TATAAAAGGA
C.lupini RB173            AAAAAGAAGG AAAAAATAG- ------AAAA CTACTACGTA AGTTCATAAT AAAGTATATA AACAATATAT TTAGAGCTTT TATAAAAGGA
C.tamarilloi RB018        AAAAAGAAGG AAAAAATAG- ------AAAA CTACTACGTA AGTTAACAAT AAAGTATATG AATAATATAT TTAGAGCTTT TATAAAAGGA
C.nymphaeae RB190         AAAGAAGAGG AAAAGAAAA- -----CGAAC TTAATACGTA GGTTAATAAT AAAATATATA AACGAAGTAT TTAGAGCTTT TATAGAACGA
C.costaricense RB184      AAAAAGAAGG AAAAAATAG- ------AAAA CTACTACGTA AGTCAATAAT AAAGTATATA AATAAAATAT TTAAAGCTTT TATAAAACGA
C.abscissum RB197         AAAACGAAGG AAAAAATAG- ------AAAT CTACTACGTA AGTCAATAGT AAAGTATATA AATAAAATAC TTAGAGCTTT TGTAAAACGA
C.simmondsii RB179        AAGGAAAAGA AAAAGAAAAA GGGAACGAAC CTAATACATA AGTTAATAAT AAAATATATA AACAAAGTAT TTAGAGCTTT TATAAAACGA

C.lupini RB221            ATTTCATACT AGCTATTAAA GCTTTTTATA TTGAGTATAC G--------- ---------- -------GAA CAAAGTATAA TAATAAAAAG
C.lupini RB173            ATTTCATACT AGCTATTAAA GCTTTTTATA TTGAGTATAC G--------- ---------- -------GAA CAAAGTATAA TAATAAAAAG
C.tamarilloi RB018        GTTTAATACT AGTTATCAAA GCTTTTTATA TCAAGCATAT A--------- ---------- -------GAA TAAAGTATAA TAATAGATAG
C.nymphaeae RB190         ACTTAAAACT AGACTTCGAA ACTTTTCATA CTAAGCATGT AGATATTACT A--------- AAAAGAGAAA CCTAATATAA TAATAA----
C.costaricense RB184      ATTTAATACT AATTACTAAA GCTTCTTATA TTAAGTATAT AAATACTAAT ATTATTTAAA AAAAGAAGAA TAAAATATAA TAATAAAAAG
C.abscissum RB197         ATTAAATATT AATTATTGAA GCTTCTTATA TTAAGTATAT AAATAGTATT T--------- --AAAAAAAG AAGAATAAAA TA-TAAAAAG
C.simmondsii RB179        ATTTAAAACT AAACCTCAAA ACTTTTCATG GTAAGTATGT AAGTATTGTT A--------- GAAAGAGAAA TATAATATGG TAATAAAAAA

C.lupini RB221            ATTATAATAG TAAAAACGGA GTTTCTCAAG TTGATTTTGA ATATTAGTAA AGTATAGAGC TGATTTCTAG ACTAGAAGCT TAATAAAGAG
C.lupini RB173            ATTATAATAG TAAAAACGGA GTTTCTCAAG TTGATTTTGA ATATTAGTAA AGTATAGAGC TGATTTCTAG ACTAGAAGCT TAATAAAGAG
C.tamarilloi RB018        ATTATAATAA TAAAAACGGA GTTCCTCAAG TTGATTTTGA ATATTGGTAA AGTATAGAGC TGATTTCTGA ATTGGAAGCT TAATAAAGAG
C.nymphaeae RB190         ---------- ---GAACGAA GTTTCTTAAG TTTATATTTA ATAAGAATAG AGCAGGGAGT TTATTTTGAT ATTAGAAGCT TTATAGAGGA
C.costaricense RB184      ATTATAGTGA TAAGAGCGGG TTTTCTTAAG TTGATTTTGA ATATTAATAG AGTATAGAGC TAGGTTTAAG ATTAGAAGCT TAATAGGGAG
C.abscissum RB197         ATTATAGTAA TAAAAACGGA GTTTTTTAAG TTTATTTTGA ATATCAGTAG AGTATAGAGC TAATTATAAG ATTGGAAGCT TAAAAGAGAG
C.simmondsii RB179        ATCATAGCAA TAAGAACGAG GTTTCTTGAG TTTATATTTA ATAAGAATAG AGTGGGGAGT TTATCTTGAT ATTAAAAGCT TTATAGAGCA

                                                                                                 ← CLR
C.lupini RB221            TTTCTTTAAA AGTATAATGT TAAATATGTA GAATTAGGCT AGTGGTACTT TAGAATGGAC GCCATAAACG CCTAAGAACC GC
C.lupini RB173            TTTCTTTAAA AGTATAATGT TAAATATGTA GAATTAGGCT AGTGGTACTT TAGAATGGAC GCCATAAACG CCTAAGAACC GC
C.tamarilloi RB018        TTTCTTTAAA AGCATAATGT TAAATATGTA GAATTAGGCC ATTGGTACTT TAGAATGGAC GCCATAAACG CCTAAGAACC GC
C.nymphaeae RB190         TTTTTTTAAA AGTGTTATAT CAAATACATA GAACAAGGCC ACTGGTGCTT TGGAATAGAC GCTGTTAACG TCTAAGAACC GC
C.costaricense RB184      TTTCTTTAAA GGTATAATGT TAAATATATA GAATTAGGCT ATTGGTGCTT TAGAATAGAC GCTATAAACG CCTGAGAACC GC
C.abscissum RB197         TTTCTTTAAA AGTATAATAT TAAATATGTA GAATTAGGCC ATTGGTGCTT TAGAGTAGAC GTTATAAACG CCTAAGAACC GC
C.simmondsii RB179        TTTCCTTAAA AGTATTGTAC CAAATACATA GAACAAGGCC ACTGGTGCTT TGGAATAGAC GCTGTCAACG TCTAAGAACC GC
```
