# Supplementary material for: Molecular Detection of the Seed-Borne Pathogen Colletotrichum lupini Targeting the Hyper-Variable IGS Region of the Ribosomal Cluster
Source: Plants (Basel). 2019 Jul 14;8(7):222. doi: 10.3390/plants8070222 (PMC6681257; doi:10.3390/plants8070222)
Supplement: Supplementary file 1 [file plants-08-00222-s001.zip › Table S1.pdf]

Table S1. DNA concentrations and absorbance ratios of *Lupinus* spp. seed batches artificially infected with *Colletotrichum lupini* IMI504893

Batch	Species	Cultivar	DNA µg µl^{-1} *	A_{260}/A_{280} **	A_{260}/A_{230} **
1:10	*L. albus*	Multitalia	32.8	1.78	2.21
	L. luteus	Mister	30.1	1.78	2.25
	L. angustifolius	Tango	36.3	1.77	2.2
1:100	*L. albus*	Multitalia	45.4	1.93	1.78
	L. luteus	Mister	10.6	1.86	1.98
	L. angustifolius	Tango	1.69	2.2	1.85
1:1000	*L. albus*	Multitalia	20.3	1.9	2.24
	L. luteus	Mister	72.1	2.01	2.28
	L. angustifolius	Tango	122	1.8	2.28
1:10000	*L. albus*	Multitalia	19.1	1.63	1.82
	L. luteus	Mister	44.7	1.87	2.04
	L. angustifolius	Tango	73.4	1.83	1.8

*To determine the concentration of the DNA solution a Qubit™ fluorometer (Invitrogen) was used according to manufacturer's protocols.

** Absorbances were estimated by a GeneQuant II spectrophotometer (Pharmacia Biotech, Cambridge, UK).
