# Supplementary figures and images for: Molecular Detection of the Seed-Borne Pathogen Colletotrichum lupini Targeting the Hyper-Variable IGS Region of the Ribosomal Cluster
Source: Plants (Basel). 2019 Jul 14;8(7):222. doi: 10.3390/plants8070222 (PMC6681257; doi:10.3390/plants8070222)

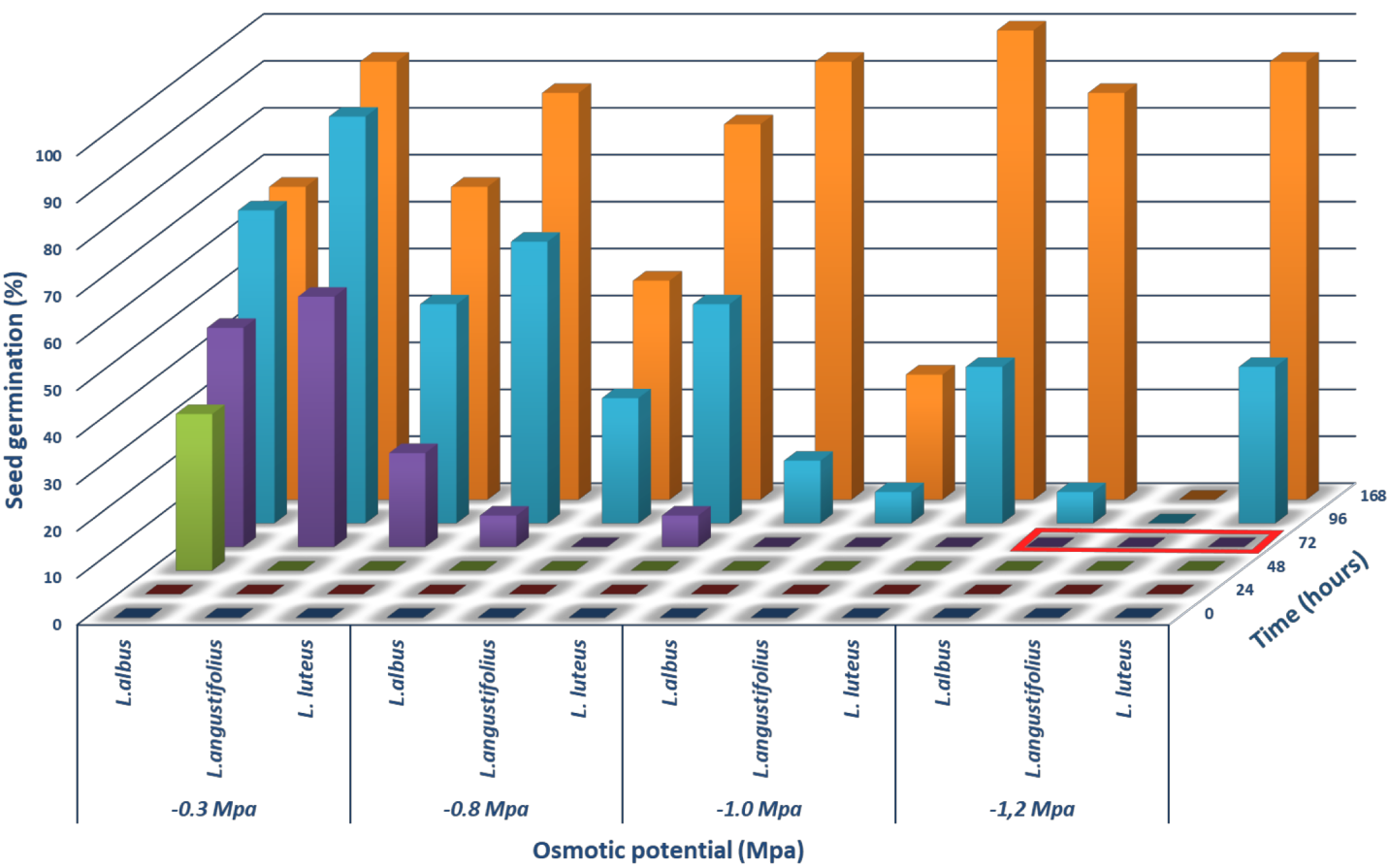

Supplement: Supplementary file 1 [file plants-08-00222-s001.zip › Figure S1.pdf]

500 bp -

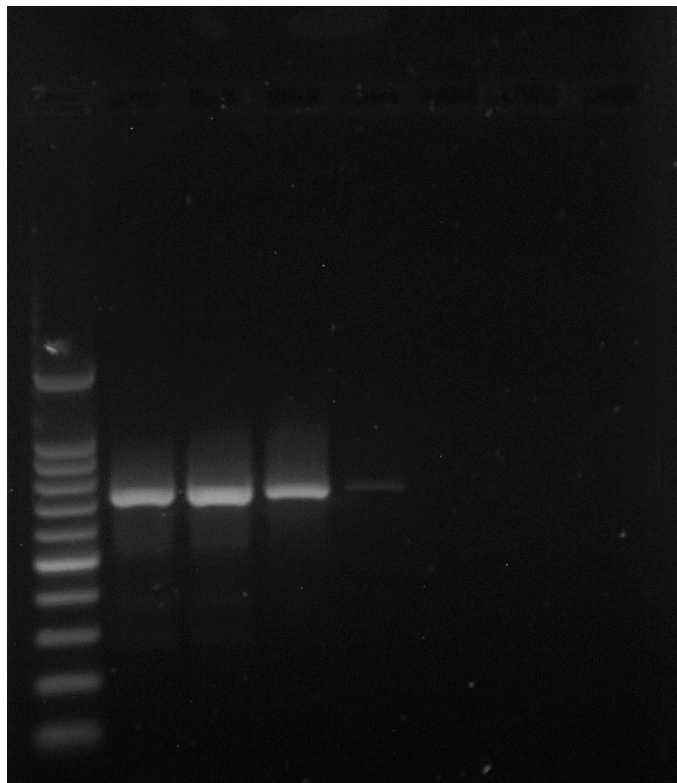

MARKER

10 ng

1 ng

0.1 ng

0.01 ng

0.001 ng

0.0001 ng

Negative ctrl

Supplement: Supplementary file 1 [file plants-08-00222-s001.zip › Figure S3v2.pdf]

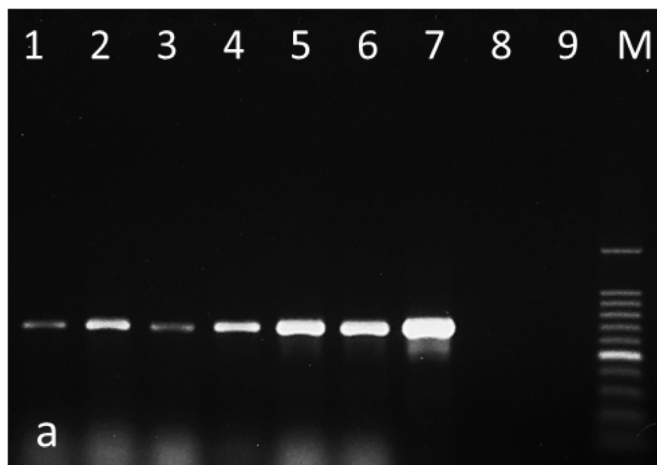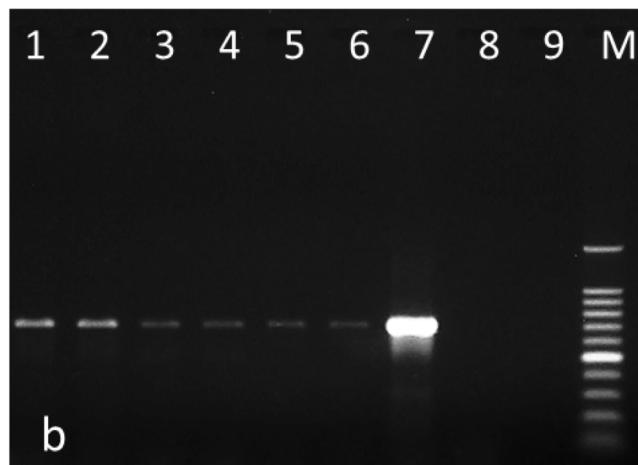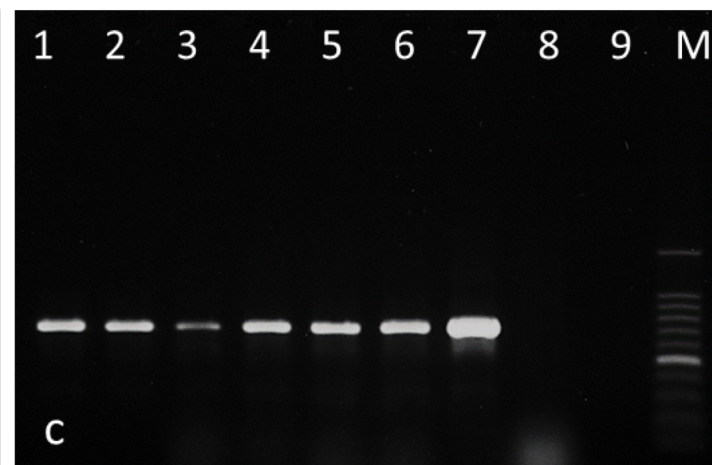

Supplement: Supplementary file 1 [file plants-08-00222-s001.zip › Figure S4v2.pdf]
